# Prognostic factors of pancreatic tumors in children and adolescents: a population study based on the surveillance, epidemiology, and end results database

**DOI:** 10.1186/s12876-024-03194-y

**Published:** 2024-03-14

**Authors:** Xianzhong Qi, Bi Zhou, Fuhua Liang, Xinxin Wang

**Affiliations:** 1 Department of Pathology, First People’s Hospital of Linping District, Hangzhou, Zhejiang China; 2 Department of Pediatrics, Suzhou Hospital of Anhui Medical University, Suzhou, Anhui China; 3 Department of Pediatric Surgery, Nanning Women and Children’s Hospital, Nanning, Guangxi China; 4 Department of Radiation Oncology, The Third Hospital of Nanchang, 330025 Nanchang, Jiangxi China

**Keywords:** Pancreatic tumors, Neuroendocrine tumors, Solid pseudopapillary tumors, Survival

## Abstract

**Purpose:**

Pancreatic tumors in children are uncommon, and data is scarce. The purpose of this study is to examine the prognostic factors of pediatric pancreatic tumors in a population-based cohort.

**Methods:**

The Surveillance, Epidemiology, and End Results (SEER) database was used to identify all pediatric patients with pancreatic tumors diagnosed between 1975 and 2018. The overall survival (OS) rates were determined using a Kaplan-Meier analysis. The log-rank test was used for univariate survival analysis. Cox proportional-hazards regression was used to determine the variables related to OS.

**Results:**

We identified 195 children with pancreatic tumors, with a median age at diagnosis of 16 years. Tumors were classified as neuroendocrine tumors (33.8%), solid pseudopapillary tumors (SPTs) (32.3%), pancreatoblastoma (11.3%), and others (22.6%). Of the patients, 30.3% had distant metastases, and 69.7% had surgery. Pancreatoblastomas were more common in younger children, whereas solid pseudopapillary tumors were more common in female patients. Overall 1-year, 3-year, and 5-year survival rates for all patients were 90.3%, 79.2%, and 77.7%, respectively. The Cox proportional hazard regression revealed that SEER stage and surgery were significant independent predictors of overall survival.

**Conclusions:**

Pancreatic tumors are rare in children, and overall survival is grim except for SPTs. SEER stage and surgery were determined to be the most relevant determinants of OS in our study.

## Introduction

Pediatric pancreatic tumors, with a prevalence estimated at 1–2 cases per ten million children and adolescents in the United States, represent a notably rare oncological entity [1–3]. The majority of our current knowledge of the incidence and prognosis of pancreatic tumors in pediatric patients is based on limited single institutional case series [4–8]. Among adults, pancreatic ductal adenocarcinoma is by far the most frequent histology [9]. On the other hand, a large range of tumors are seen in children. Solid pseudopapillary tumors (SPTs) are thought to be the most prevalent malignancy in the second decade of life, whereas pancreatoblastoma is considered to be the most common during the first decade of life [10–11]. In addition, neuroendocrine tumors, epithelial carcinomas and sarcomas have also been reported in children [12–13]. The rarity and diversity of these malignancies, coupled with the absence of standardized treatment protocols, present significant challenges in clinical research and management.

To the best of our knowledge, however, given the rarity of pancreatic tumors, very few sizable cohort studies have explored the epidemiology and survival outcomes of patients with these tumors. Therefore, utilizing data from a sizable population-based database, the current study aimed to more thoroughly identify and assess the prognostic factors among children with pancreatic tumors.

## Methods

### Study population

Children and adolescents (age ≤ 19 years) diagnosed with primary pancreatic tumors between 1975 and 2018 were included in the study. For pancreatic tumors, we used the Third Edition (ICD-O-3) morphological code (pseudopapillary neoplasm: 8452; pancreatoblastoma: 8971; endocrine tumors: 8240, 8246, 8150; sarcomas: 9930, 9755; pancreatic adenocarcinoma: 8140; acinar cell carcinoma: 8550). Using the “case listing” option, demographic parameters were extracted. Three categories were used to categorize tumor grades: low or moderately differentiated, poorly or undifferentiated, and unknown. The SEER historic stage was used to determine the staging information. Localized disease is restricted to the primary organ and shows no signs of spreading; regional disease has spread to surrounding tissues or lymph nodes; and distant tumors have spread from the primary site to distant organs or lymph nodes. The diagnosis age ranges were divided into two groups: 0–14 years old and 15–19 years old. Four types of treatments were used to categorize the cohorts: no treatment, surgery alone, chemotherapy alone, and surgery plus chemotherapy. Only patients with pathological confirmation were included in this study. Patients with unknown important data, such as SEER stage, surgery status and incomplete follow-up information were excluded. Informed consent was waived because the data were gathered from a public database.

### Statistical analysis

All statistical analyses were performed using SPSS software (version 22.0, SPSS Inc., Chicago, IL, USA). The chi-square test was used to compare the frequency distribution of categorical variables. Our primary outcome was overall survival (OS), which was calculated from the date of diagnosis to the date of death. To estimate survival curves across treatments, the Kaplan-Meier method was utilized, and the log-rank test was performed to compare survival rates. The Cox proportional hazards model was used to determine mortality hazard ratios (HR) with 95% confidence intervals (CI). Variables with a *P* < 0.10 in univariate analysis were selected for multivariate analysis. The statistical significance was determined using a two-tailed P-value of 0.05.

## Results

### Patient characteristics

A total of 195 children and adolescents with malignant pancreatic tumors were identified from 1975 to 2018. The median patient age was 16 years (range: 1–19 years). The majority of patients (74.9%) were white and female (65.7%). Eighty-four patients (43.0%) were ≤ 14 years old, while 111 (57.0%) were 15–19 years old. Distant metastases were found in 30.3% of the patients, whereas 48.7% (95/195) and 21% (41/195) had localized and regional disease, respectively. Only 66 cases had tumor grade information; 28.2% had well or moderate differentiation, while 5.6% had poorly or undifferentiated tumors. In terms of treatment, the majority of them were treated with surgery (69.7%) and without chemotherapy (69.2%). Eighty-three (42.6%) had lymph nodes removed. Furthermore, surgery alone was the most often used treatment regimen (58.5%).

Table 1 shows the 195 patients with validated pancreatic tumor subtypes. The most prevalent type of tumor was neuroendocrine tumor (33.8%), followed by solid pseudopapillary tumor (32.3%). Pancreatoblastoma was more common in younger children, whereas solid pseudopapillary tumors were more common in female patients (*p* < 0.001). The proportion of localized stage (71.4%), surgery (93.7%) and lymph nodes removed (69.8%) were highest in solid pseudopapillary tumors.

**Table 1:** Basic characteristics of patients with pancreatic tumors stratified by tumor histology

Characteristics	SPT	NET	PB	Others	P value
Age at diagnosis, n (%)					< 0.001
≤ 14-year-old	21 (33.3%)	19 (28.8%)	20 (90.9%)	24 (54.5%)	
> 14-year-old	42 (66.7%)	47 (71.2%)	2 (9.1%)	20 (45.5%)	
Gender, n (%)					< 0.001
Female	57 (90.5%)	35 (53%)	10 (45.5%)	26 (59.1%)	
Male	6 (9.5%)	31 (47%)	12 (54.5%)	18 (40.9%)	
Race, n (%)					0.569
White	47 (74.6%)	50 (75.8%)	14 (63.6%)	35 (79.5%)	
Others	16 (25.4%)	16 (24.2%)	8 (36.4%)	9 (20.5%)	
Primary sites, n (%)					0.024
Head	21 (33.3%)	19 (28.8%)	8 (36.4%)	25 (56.8%)	
Body	7 (11.1%)	5 (7.6%)	2 (9.1%)	6 (13.6%)	
Tail	21 (33.3%)	17 (25.8%)	4 (18.2%)	3 (6.8%)	
Unknown	14 (22.2%)	25 (37.9%)	8 (36.4%)	10 (22.7%)	
Grade, n (%)					< 0.001
Low/moderate (I/II)	20 (31.7%)	28 (42.4%)	0 (0%)	7 (15.9%)	
Poor/undifferentiated (III/IV)	1 (1.6%)	4 (6.1%)	1 (4.5%)	5 (11.4%)	
Unknown	42 (66.7%)	34 (51.5%)	21 (95.5%)	32 (72.7%)	
Tumor size (cm), n (%)					0.065
≤ 5	7 (11.1%)	17 (25.8%)	4 (18.2%)	10 (22.7%)	
> 5	19 (30.2%)	9 (13.6%)	5 (22.7%)	4 (9.1%)	
Unknown	37 (58.7%)	40 (60.6%)	13 (59.1%)	30 (68.2%)	
SEER stage, n (%)					< 0.001
Localized	45 (71.4%)	29 (43.9%)	7 (31.8%)	14 (31.8%)	
Regional	16 (25.4%)	10 (15.2%)	4 (18.2%)	11 (25%)	
Distant	2 (3.2%)	27 (40.9%)	11 (50%)	19 (43.2%)	
Surgery, n (%)					< 0.001
Yes	59 (93.7%)	38 (57.6%)	16 (72.7%)	23 (52.3%)	
No	4 (6.3%)	28 (42.4%)	6 (27.3%)	21 (47.7%)	
Lymph nodes removed, n (%)					< 0.001
Yes	44 (69.8%)	26 (39.4%)	6 (27.3%)	7 (15.9%)	
No	19 (30.2%)	40 (60.6%)	16 (72.7%)	37 (84.1%)	
Chemotherapy, n (%)					< 0.001
Yes	2 (3.2%)	20 (30.3%)	11 (50%)	27 (61.4%)	
No	61 (96.8%)	46 (69.7%)	11 (50%)	17 (38.6%)	
Treatment regimens, n (%)					< 0.001
No treatment	4 (6.3%)	11 (16.7%)	2 (9.1%)	4 (9.1%)	
Surgery alone	57 (90.5%)	35 (53%)	9 (40.9%)	13 (29.5%)	
Chemotherapy alone	0 (0%)	17 (25.8%)	4 (18.2%)	17 (38.6%)	
Surgery plus chemotherapy	2 (3.2%)	3 (4.5%)	7 (31.8%)	10 (22.7%)	

### Survival analysis

**Fig. 1:**
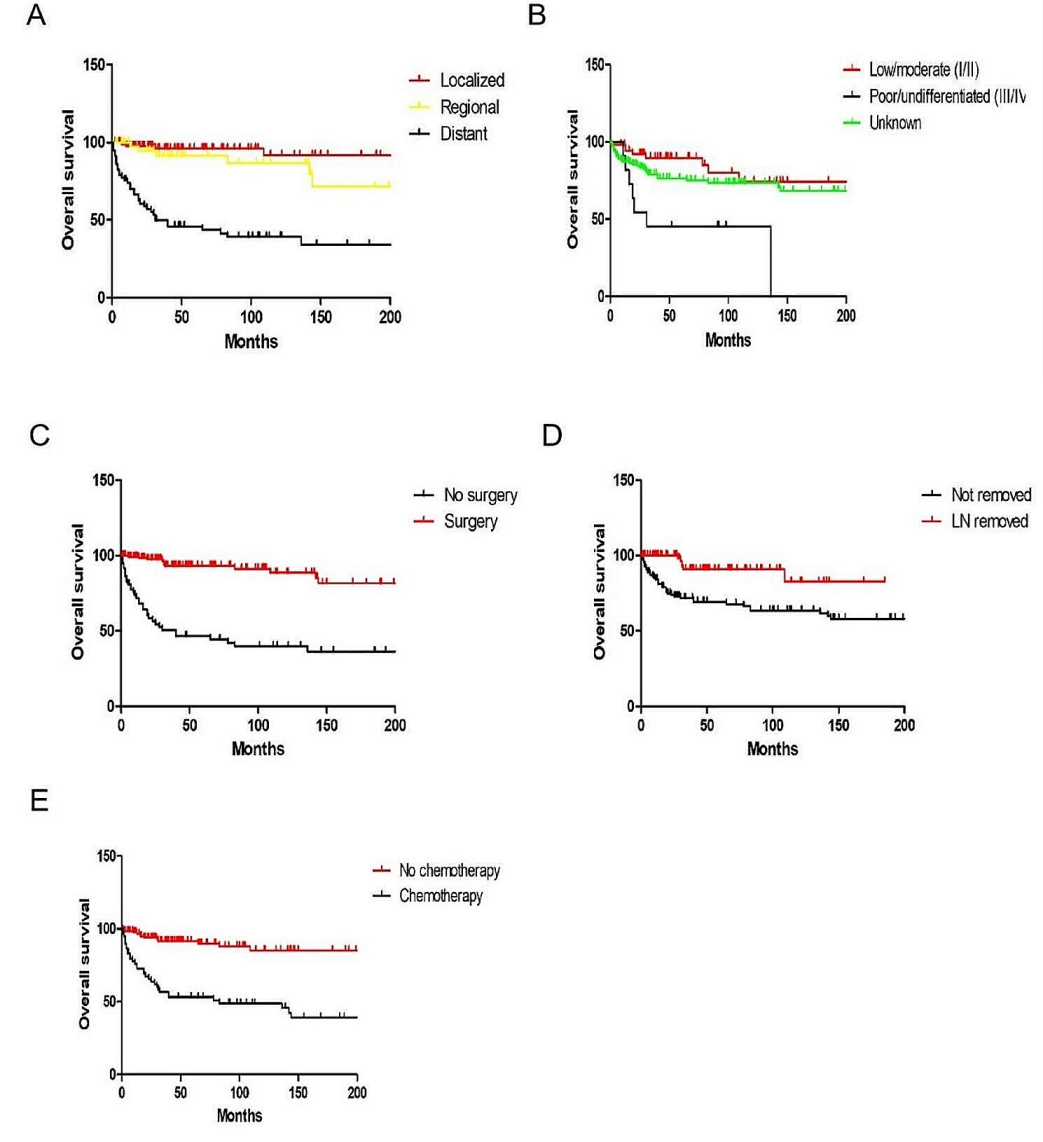
Kaplan–Meier analysis of OS in pediatric pancreatic tumors stratified by the SEER stage, grade, surgery, lymph nodes (LN) removed and chemotherapy. (**A**) Distant vs. Localized and regional, *P* < 0.001. (**B**) Low and moderately differentiated vs. Poorly and undifferentiated, *P* = 0.013. (**C**) Surgery vs. No surgery, *P* < 0.001. (**D**) LN removed vs. LN not removed, *P* = 0.001. (**E**) Chemotherapy vs. No chemotherapy, *P* < 0.001

**Fig. 2:**
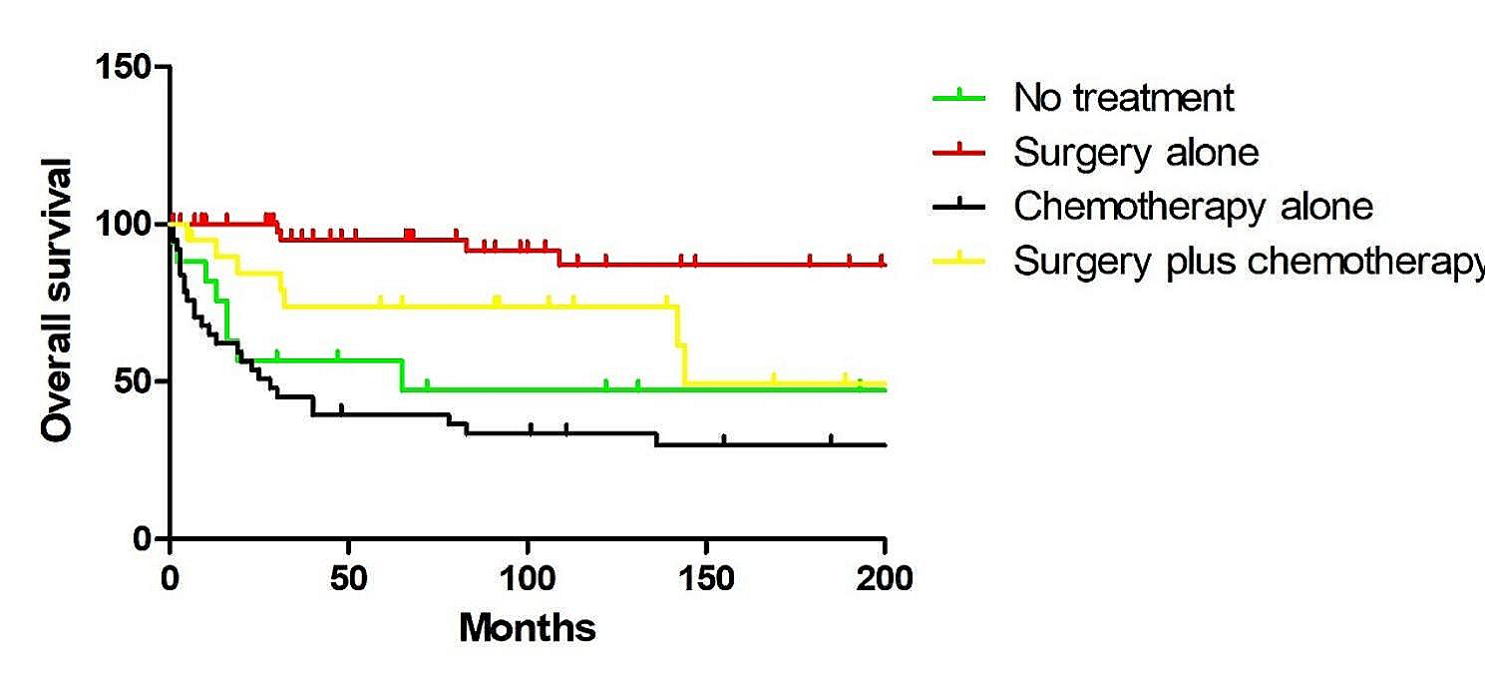
Kaplan–Meier analysis of OS in pediatric pancreatic tumors except SPT, when stratified by chemotherapy and surgery. Surgery-based regimens were associated with significantly improved the 5-y OS rate compared with other treatment regimens (*P* < 0.001)

All patients had overall survival rates of 90.3% at 1- year, 79.2% at 3- year, and 77.7% at 5- year. Regarding age (*P* = 0.952) and race (*P* = 0.878), there was no significant difference. Additionally, no statistically significant variation in OS was seen according to the primary sites (*P* = 0.268), tumor size (*P* = 0.797) and histology (*P* = 0.996). Nonetheless, there was a substantial gender difference in survival rates, with female patients having superior outcomes (*P* = 0.044). Individuals with local disease had a longer survival rate than those with distant illness (*P* < 0.001) (Fig. 1A). Moreover, patients with low or moderate grade had a better survival than those with poor or undifferentiated grade (*P* = 0.013) (Fig. 1B). Histologically, patients with solid pseudopapillary tumors had the greatest OS. Regarding therapy, patients who underwent surgery outlived non-surgery patients (*P* < 0.001) (Fig. 1C), and patients who received lymph nodes removed also had better survival (*P* = 0.001) (Fig. 1D). However, patients who received chemotherapy had poorer survival than patients who didn’t (*P* < 0.001) (Fig. 1E). Surgery-based regimens produced the highest survival results when compared to other regimens (Fig. 2).

### Prognosis analysis

The results of multivariate analysis using the Cox regression model are summarized in Table 2. In this model, multivariate analysis of the entire cohort revealed that SEER stage and surgery were significant independent predictors of overall survival. The risk of death was higher for distant disease (hazard ratio (HR) 7.88, 95% confidence interval (CI), 2.67–23.20; *P* < 0.001) as compared to localized disease. Furthermore, multivariate analysis demonstrated a substantial level of protection with surgery (HR 3.0, 95% CI, 1.24–7.27; *P* = 0.015).

**Table 2:** Survival analyses of overall survival for pediatric and adolescent pancreatic tumors

Characteristics	Total(N)	Univariate analysis			Multivariate analysis	
		Hazard ratio (95% CI)	P value		Hazard ratio (95% CI)	P value
Age at diagnosis	195					
> 14-year-old	111	Reference				
≤ 14-year-old	84	0.982 (0.547–1.762)	0.952			
Gender	195					
Female	128	Reference			Reference	
Male	67	1.803 (1.016–3.200)	0.044		1.035 (0.559–1.918)	0.912
Race	195					
Others	49	Reference				
White	146	0.951 (0.500–1.809)	0.878			
Grade	195					
Unknown	129	Reference			Reference	
Low/moderate (I/II)	55	0.617 (0.293–1.301)	0.205		1.275 (0.545–2.981)	0.575
Poor/undifferentiated (III/IV)	11	2.877 (1.255–6.593)	0.013		1.301 (0.543–3.118)	0.555
Tumor size (cm)	195					
≤ 5	38	Reference				
> 5	37	0.769 (0.244–2.424)	0.654			
Unknown	120	1.113 (0.490–2.528)	0.797			
SEER stage	195					
Localized	95	Reference			Reference	
Regional	41	3.456 (1.010–11.817)	0.048		4.090 (1.134–14.758)	0.031
Distant	59	16.710 (5.944–46.977)	< 0.001		7.877 (2.675–23.197)	< 0.001
Surgery	195					
Yes	136	Reference			Reference	
No	59	7.810 (4.028–15.141)	< 0.001		3.005 (1.241–7.274)	0.015
Lymph nodes removed	195					
Yes	83	Reference			Reference	
No	112	3.830 (1.701–8.625)	0.001		1.541 (0.620–3.829)	0.351
Chemotherapy	195					
No	135	Reference			Reference	
Yes	60	5.592 (2.932–10.665)	< 0.001		1.471 (0.672–3.216)	0.334

## Discussion

Pancreatic tumors are rare in children and are inadequately reported in the literature [14–15]. Utilizing data from the SEER database, our study provides a comprehensive population-based analysis of pediatric pancreatic tumors spanning from 1975 to 2018. Predominant histologies identified in our cohort included neuroendocrine tumors, accounting for 33.8%, and solid pseudopapillary tumors, comprising 32.3%. We also found pancreatoblastoma was more frequently diagnosed in younger children, while solid pseudopapillary tumors predominated among female patients. In cases of solid pseudopapillary tumors, the rates of localized stage, surgery, and lymph node removal were notably higher. In this study, both the univariate and multivariable analyses revealed that SEER stage and surgery were statistically significant factors associated with overall survival.

Although there is evidence that males with solid pseudopapillary neoplasm may have an atypically aggressive disease [16], this was not proven in the present investigation. Our study found that patients with solid pseudopapillary tumors had the best overall survival for both sexes (5-year OS was 100%). Furthermore, our study highlights the significance of tumor stage as a prognostic indicator. The 5-year survival rate for localized tumors was 95.9%, while the 5-year survival rate for distant tumors was 45.2%. This study also revealed that gender was a crucial predictor of survival, with female patients exhibiting a significantly greater survival rate than male counterparts, which was consistent with the previous study [17]. However, given that solid pseudopapillary tumors are known to have a better biology and a less aggressive clinical course than other malignant neoplasms, we hypothesized that the significantly higher incidence of this tumor type in females may account for the significantly better survival rates we saw in our series of female patients. After excluding the solid pseudopapillary tumor from the survival analysis, we indeed discovered that the difference in 5-year overall survival between males and females was no longer significant.

The role of complete surgical resection in the management of localized, resectable pancreatic tumors is underscored [18]. The primary tumor and any surrounding lymph nodes or tissues that are impacted are to be removed during surgery. As the current standard of care, total primary resection and debulking of metastatic lesions are advised in certain tumor forms (solid pseudopapillary tumors, endocrine tumors, pancreatoblastoma) [19–21]. Consequently, it is not unexpected that patients who underwent surgery had much higher survival rates. Surgical resection was linked to longer survival on multivariate analysis (hazard ratio (HR) 7.88, 95% confidence interval (CI), 2.67–23.20). However, because of selection bias, surgery was predominantly performed in situations of early stage or favorable biology, it is uncertain if surgical intervention or simply being eligible for surgery is genuinely connected with improved survival. While patients who have had their lymph nodes removed seem to have a better prognosis after this treatment, it should be emphasized that the significantly enhanced survival may be due to the procedure’s high prevalence in solid pseudopapillary tumors. In our analysis, chemotherapy was associated with significantly lower overall survival. This could be related to the increased use of chemotherapy in palliative care, as well as patients with advanced pancreatic tumors being unable to undergo surgery.

This study has some limitations that should be noted. Firstly, only 30% of the US population was included in the SEER database, and the database lacks information on family history or inherited disorders, both of which are known to increase the risk of pancreatic tumors. Similarly, the absence of clinical data (such as biomarkers or biopsy results) could have aided in explaining the treatment strategy. Secondly, while information on the surgical resection recipient was accessible, details on the degree of surgical resection were not. Finally, there is no recurrence information in the database, which could have helped define oncologic outcomes for this patient cohort. Therefore, further research is warranted to validate our findings.

In conclusion, pancreatic tumors represent a rare and diverse subset of neoplastic diseases in children. Neuroendocrine tumors were the most common histologic type while solid pseudopapillary tumors exhibited the most favorable prognosis. The most important predictors of survival were the SEER stage and surgery.

## Data Availability

The datasets generated and/or analyzed during the current study are available in the SEER database, https://seer.cancer.gov/.

## References

[1] Perez EA, Gutierrez JC, Koniaris LG (2009). Malignant pancreatic tumors: incidence and outcome in 58 pediatric patients. J Pediatr Surg.

[2] Picado O, Ferrantella A, Zabalo C (2020). Treatment patterns and outcomes for pancreatic tumors in children: an analysis of the National Cancer Database. Pediatr Surg Int.

[3] Brecht IB, Schneider DT, Klöppel G (2011). Malignant pancreatic tumors in children and young adults: evaluation of 228 patients identified through the Surveillance, Epidemiology, and end result (SEER) database. Klin Padiatr.

[4] Nasher O, Hall NJ, Sebire NJ (2015). Pancreatic tumours in children: diagnosis, treatment and outcome. Pediatr Surg Int.

[5] Yu DC, Kozakewich HP, Perez-Atayde AR (2009). Childhood pancreatic tumors: a single institution experience. J Pediatr Surg.

[6] Rojas Y, Warneke CL, Dhamne CA (2012). Primary malignant pancreatic neoplasms in children and adolescents: a 20 year experience. J Pediatr Surg.

[7] Tian D, Zhu H, Wei X (2022). Pancreaticoduodenal and choledochal hemangiomatosis with vascular variation in a child: a rare disease with challenge starts from diagnosis-a case report. World J Surg Oncol.

[8] Hu MG, Xiao YH, Song DD (2017). First experience of robotic spleen-preserving distal pancreatectomy in a child with insulinoma. World J Surg Oncol.

[9] Cao L, Huang C, Cui Zhou D (2021). Proteogenomic characterization of pancreatic ductal adenocarcinoma. Cell.

[10] Dall’igna P, Cecchetto G, Bisogno G (2010). Pancreatic tumors in children and adolescents: the Italian TREP project experience. Pediatr Blood Cancer.

[11] Mylonas KS, Nasioudis D, Tsilimigras DI (2018). A population-based analysis of a rare oncologic entity: malignant pancreatic tumors in children. J Pediatr Surg.

[12] Shorter NA, Glick RD, Klimstra DS (2002). Malignant pancreatic tumors in childhood and adolescence: the Memorial Sloan-Kettering experience, 1967 to present. J Pediatr Surg.

[13] Park M, Koh KN, Kim BE (2011). Pancreatic neoplasms in childhood and adolescence. J Pediatr Hematol Oncol.

[14] Sacco Casamassima MG, Gause CD, Goldstein SD (2016). Pancreatic surgery for tumors in children and adolescents. Pediatr Surg Int.

[15] Vossen S, Goretzki PE, Goebel U (1998). Therapeutic management of rare malignant pancreatic tumors in children. World J Surg.

[16] Lin MY, Stabile BE (2010). Solid pseudopapillary neoplasm of the pancreas: a rare and atypically aggressive disease among male patients. Am Surg.

[17] He Y, Su Y, Zeng J et al. Cancer-specific survival after diagnosis in men versus women: A pan-cancer analysis. MedComm (2020). 2022; 3(3): e145. 10.1002/mco2.145 PMC9246337 35783087

[18] Karunakaran M, Barreto SG (2021). Surgery for pancreatic cancer: current controversies and challenges. Future Oncol.

[19] Martin RC, Klimstra DS, Brennan MF (2002). Solid-pseudopapillary tumor of the pancreas: a surgical enigma?. Ann Surg Oncol.

[20] Kang CM, Kim KS, Choi JS (2006). Solid pseudopapillary tumor of the pancreas suggesting malignant potential. Pancreas.

[21] Feng T, Lv W, Yuan M (2019). Surgical resection of the primary tumor leads to prolonged survival in metastatic pancreatic neuroendocrine carcinoma. World J Surg Oncol.

